# Polyphenol Extracts from Grape Seeds and Apple Can Reactivate Latent HIV-1 Transcription through Promoting P-TEFb Release from 7SK snRNP

**DOI:** 10.1155/2022/6055347

**Published:** 2022-02-07

**Authors:** Cong Wang, Huiru Wang, Zhenrui Pan, Jun Wu, Yafei Guo, Jing Zhang, Zixun Xiang, Wei Lu, Yuhua Xue

**Affiliations:** ^1^School of Pharmaceutical Sciences and Fujian Provincial Key Laboratory of Innovative Drug Target Research, Xiamen University, Xiamen, Fujian 361005, China; ^2^Medical Center of Hematology, The Second Affiliated Hospital, Army Medical University, Chongqing, China; ^3^Department of Pharmacology, Baotou Medical College, Baotou, Inner Mongolia 014040, China; ^4^Rehabilitation Hospital of Huishan, Wuxi, Jiangsu 214181, China

## Abstract

The principal barrier for the eradication of HIV/AIDS is the virus latency. One of the effective strategies so called “shock and kill” is to use latency-reversing agents (LRAs) to activate the latent HIV reservoirs and then combine them with the highly active antiretroviral therapy (HAART) to eradicate the virus. However, most of the current LRAs are too toxic; therefore, they have not been used clinically. Our preliminary data indicated that polyphenols from grape seeds can activate HIV in latently infected Jurkat T cells. Owing to a lot of food containing polyphenols and based on a reasoning whether all of these kinds of polyphenols contain the latency-reversing function, in this study, we screened 22 fruits/vegetables to see whether polyphenols from these can reactivate latent HIV-1 transcription. We finally proved that the polyphenols from grape seeds, apple, pomegranate, and bilberry can reactivate latent HIV-1 transcription. The activation of which can be detected on the level of protein and mRNA. The activation of which is in a dose- and time-dependent manner, while the activated polyphenol extracts have the effects to stimulate Tat-independent HIV-1 transcription. The mechanism shows that polyphenol extracts from grape seeds and apple can stimulate P-TEFb's release from 7SK snRNP to induce HIV gene transcription. These results indicate that using a few food of high-content polyphenols as latent activators and combining HARRT may be of great use for the treatment of HIV/AIDS in the future.

## 1. Introduction

HIV which was first isolated in 1983 will attack T cells, disable the immune system, and eventually lead to HIV/AIDs [1–3]. The emergence of HIV has been a threat to public health, and it is a big challenge for human to eliminate virus because of HIV pathogenesis [4]. An important breakthrough in the area of HIV treatment is the highly active antiretroviral therapy (HAART) which has been used to reduce the levels of plasma HIV RNA below the limits of detection.

However, the treatments must be maintained for life, which can lead to serious chronic consequences and extraordinary financial constraint on the overburdened health care system [5–7]. Moreover, the interruption of HAART inevitably causes a rapid rebound of viremia [8]. This is because various cellular reservoirs, with memory CD4+ T cells being the most well-defined one, harbor integrated and transcriptionally silent proviruses, which remain replication-competent despite extended HAART [9–11]. Clearly, the HAART-mediated viral suppression alone cannot eradicate HIV, and novel approaches must be designed to eliminate the latent reservoirs.

For making up for the shortfall, several new methods urgently need to be designed to address HIV infection [12–14]. One of the potential therapeutic approaches which was called “shock and kill” showed up. The “shock” phase is aimed at reactivating the latent viral reservoirs in chronically infected individuals. Next, in the “kill” phase, the highly active antiretroviral therapy (HAART), immune system, and viral cytopathogenicity are used for destroying the infection [7, 15–17]. If the “shock and kill” strategy can be applied successfully, a critical step is to screen potential compounds to reactivate latent HIV expression [18]. Up to now, some reported latency-reversing agents such as JQ1 [19], prostratin [20], AV6 [21], PKC activators [22], and positive transcription elongation factor (P-TEFb) inducers [23] play a role in different ways. However, not only do these agents have strong toxicity and side effects but also a majority of them cannot be administered effectively for the human body [16]. Hence, we need to focus on some novel and safe latency activators to attend the process of HIV/AIDS eradication.

It is well known that a major rate-limiting step controlling HIV viral gene expression is promoter proximal pausing of initiated RNA polymerase II (Pol II) on integrated HIV proviral DNA in the elongation phase [19, 24–26]. To overcome this restriction, the HIV-encoded specific Tat protein recruits the human Super Elongation Complex (SEC) to paused Pol II through forming a multicomponent complex that also contains P-TEFb, ELL2, the TAR RNA, and a stem-loop structure located at the 5′ end of all nascent HIV transcripts [25, 27]. Once recruited to the viral promoter by Tat and TAR, P-TEFb and ELL2 synergistically activate Pol II elongation to produce full-length HIV transcripts by different mechanisms. Active P-TEFb can phosphorylate Ser2 of Pol II CTD and two negative elongation factors NELF and DSIF to stimulate transcription elongation. And ELL2 can directly stimulate the processivity of Pol II through suppressing its transient pausing. Therefore, depending on Tat to deliver P-TEFb to the paused Pol II is an essential part for HIV gene transcription [25, 28, 29].

With the development of pharmaceutical industry, dietary therapy [30, 31] as a novel method attracts the general public's attention. Nowadays, dietary therapy not only has been used in the treatment of the disease but also gives rise to the application of some health care products because this method has provided great benefits for human health [32–34]. Previously, our laboratory proved that procyanidin C-13,3′,3^″^-tri-O-gallate (named as REJ-C1G3) from *Polygonum cuspidatum* Sieb. et Zucc which belongs to polyphenols can activate HIV transcription in latently infected Jurkat T cells [26]. And in recent years, great attention is focused on the research of polyphenols including epigallocatechin-3-gallate, quercetin, resveratrol, and myricetin in the academic field, regarding their different functions such as anticancer and antioxidant [35]. Outcomes of some researches have indicated that often taking in polyphenol-rich food like fruits has a powerful role in strengthening the human body's ability to prevent disease [36, 37]. According to the above contents and based on a reasoning whether all of these kinds of polyphenols contain the latency-reversing function, this present article points out that the polyphenols from grape seeds [35, 36, 38] possess the activation which can be proven on the level of protein and mRNA though treating different cell lines. What is more, we proved that other such similar polyphenol-rich foods, like apple polyphenols [39, 40], pomegranate polyphenols [41], and bilberry polyphenols [42, 43], show a consistent result compared with polyphenols from grape seeds. All the results indicate that in combination with a few food of high-content polyphenols as latent activators and HARRT, it may be a promising strategy formed by dietary therapy and chemotherapeutics in curing HIV/AIDS and of great use for public health in the future.

## 2. Materials and Methods

### 2.1. Extract Materials

Polyphenol extracts from grape seeds, pomegranate, bilberry (GNC, USA), and apple polyphenols (Yuanye Biology Company, Shanghai, China) and the other polyphenolic extracts (Changyue Biology Company, Xi'an, China) were purchased from commercial resources. These polyphenol-rich extracts were dissolved with DMSO in the process of experiments.

### 2.2. Antibodies

The anti-GFP (RLM3124; Ruiying Biological) and anti-*α*-tubulin (T6074; Sigma-Aldrich) were used in the western blotting.

### 2.3. Flow Cytometry for Activity Screening

J-Lat A2 cells [44] were treated with polyphenol extracts from grape seeds, apple, pomegranate, bilberry, and other foods at the indicated concentrations and times in the figure or figure legends. After the treatment, cells were harvested at 2000 rpm for 5 min, washed in cold phosphate-buffered saline (PBS) and centrifuged at the same centrifugal condition, and resuspended and filtered in PBS. The outcomes were determined by flow cytometry (FACS) (Beckman Coulter, Miami, FL, USA). 2D10 cells [45] were treated and analyzed like J-Lat A2 cells.

### 2.4. Quantitative PCR

The reactions were treated with the Applied Biosystems Real-Time PCR System and CWBIO Ultra SYBR Mixture RT-PCR reagents according to the manufacturers' instructions. The sequences of the primers used in PCR are as follows: EGFP-F: CAGTGCTTCAGCCGCTACCC; EGFP-R: AGTTCACCTTGATGCCGTTCTT; GAPDH-F: GCACCACCAACTGCTTAGC; and GAPDH-R: GGCATGGACTGTGGTCATG. PCR conditions included an initial denaturing step at 95°C for 10 min and then 40 cycles of amplification. Each cycle consisted of 15 sec at 95°C and 1 min at 62°C. The values were normalized to those of GAPDH to obtain the relative folds of induction [26].

### 2.5. Luciferase Assay

For the luciferase assay, the HeLa-based NH1 cells [19, 26] containing an integrated HIV-1 LTR-luciferase reporter construct but expressing no Tat, as well as its derivative NH2 cells [26], which also harbor an integrated Tat-HA expression vector, were used. Cells were then treated with different concentrations of extracts for 24 h and subjected to the luciferase assay using kit E1501 from Promega and following the manufacturer's instructions. Lysates were prepared from an approximately equal number of cells and normalized based on their contained *α*-tubulin levels among all the samples.

### 2.6. Cell Viability

Measurement of cell viability was performed with the Promega CellTiter-Glo kit according to the manufacturer's instructions. Cells were seeded at 5000 cells/well in a 96-well plate (3 wells/sample) and then treated or untreated. The measurements were taken to track cell proliferation during the course of the treatment.

## 3. Results

### 3.1. Polyphenol Extracts from Grape Seeds, Apple, Pomegranate, and Bilberry Facilitate Latent HIV Transcription

To screen agents which make latent HIV reactivation, the J-Lat A2 cell line generated by the Verdin Laboratory has been used [44]. This cell line was created by transducing an HIV vector expressing Tat-Flag and the enhanced green fluorescent protein (GFP) HIV vector under the control of the viral 5′-LTR and an internal ribosome entry site (IRES) placed in between Tat and GFP (LTR-Tat-Flag-IRES-EGFP) in human Jurkat T cells [19, 44]. In the context of activation, transcription will be performed, followed by testing GFP though flow cytometry, and we can detect whether they contain the activity. J-Lat A2 cells were incubated individually with these agents for 24 h as shown in Table 1. On the basis of results obtained by FACS, polyphenol extracts from grape seeds, pomegranate, bilberry, and apple were found to cause HIV LTR-driven GFP production in these experiments.

According to Table 1, it is important that the same consequence of these products was detected in the 2D10 cell line [19, 45], another Jurkat-based postintegrative latency cell model, which consisted of almost all the HIV genome encoding a partially attenuated Tat variant H13L and the short-lived d2EGFP protein in place of the nef gene.

**Figure 1:**
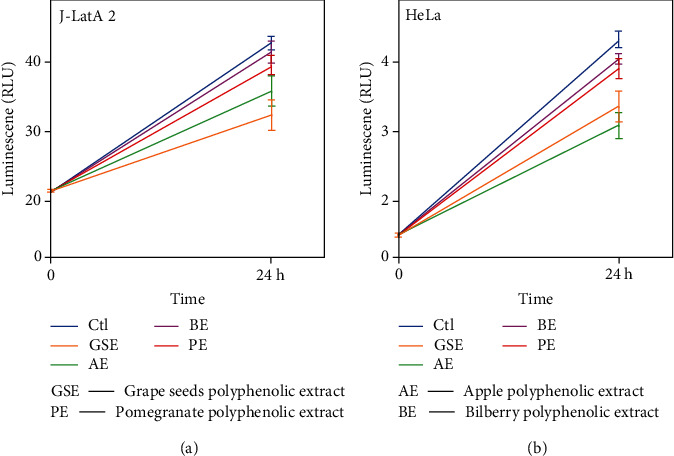
Polyphenol extracts from grape seeds, apple, pomegranate, and bilberry impact on cell viability. J-Lat A2 (a) and HeLa cells (b) were exposed to these agents (200 *μ*M) for 24 hours and then assayed for cell viability. The error bars represent mean ± SD from three independent experiments.

To evaluate the antiproliferative activity of these compounds against J-LatA2 cells, we took cell viability assays to measure the activity. The result indicated that these compounds showed the antiproliferative activity against J-Lat A2 cells and HeLa cells treated with 200 *μ*M as indicated in Figure 1. Since these compounds' treatment exceeded 200 *μ*M in all the experiments performed in the current study, we concluded that the effect of these compounds on proviral activation is not due to any significant change to cell growth and viability that may result from long-term exposure to these compounds.

### 3.2. Polyphenol Extracts from Grape Seeds, Apple, Pomegranate, and Bilberry Facilitate Latent HIV Transcription Indicated by HIV-1 LTR-Driven EGFP Expression Levels in a Dose- and Time-Dependent Manner

**Figure 2:**
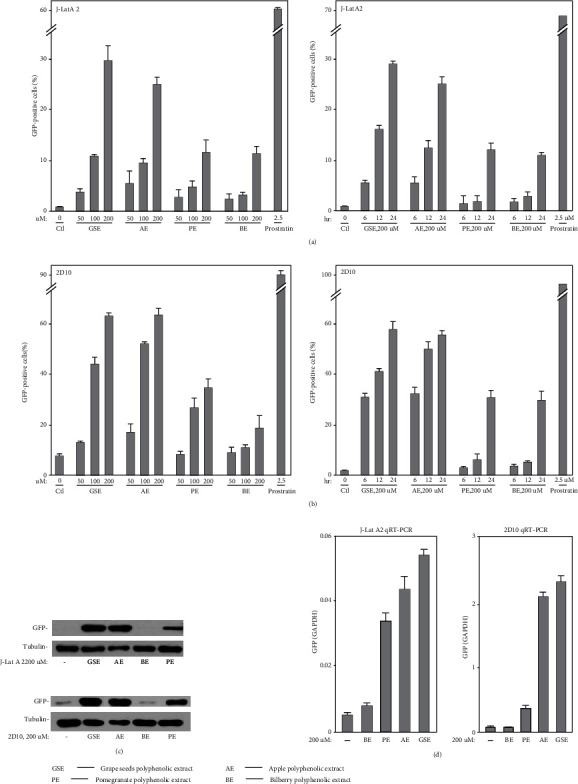
Polyphenol extracts from grape seeds, apple, pomegranate, and bilberry facilitate latent HIV transcription indicated by HIV-1 LTR-driven GFP expression levels in a dose- and time-dependent manner. (a, b) J-Lat A2 cells/2D10 cells were treated with 50 *μ*M, 100 *μ*M, and 200 *μ*M and for increasing time periods (6, 12, and 24 h) as indicated. Polyphenol extracts from grape seeds, apple, pomegranate, and bilberry and 2.5 *μ*M prostratin as a positive control for 24 h. The GFP-positive cells were detected by flow cytometry (FACS), and the percentages of GFP-expressing cells in the entire population were indicated. (c) WCE (whole cell extracts) of J-Lat A2 cells/2D10 cells incubated with 200 *μ*M of polyphenol extracts from grape seeds, apple, pomegranate, and bilberry for 24 h were analyzed by western blotting to test the content of GFP protein. (d) J-Lat A2 cells/2D10 cells were treated with the same indicated concentrations and for indicated time periods. The ratios of the GFP/GAPDH mRNA levels in treated J-Lat A2 cells/2D10 cells were determined by quantitative RT-PCR (qRT-PCR). The error bars in all panels represent mean ± SD from three independent experiments.

To test the activities of polyphenol extracts from grape seeds, apple, pomegranate, and bilberry, we treated J-Lat A2 cells with increasing concentrations of these compounds and also for different time periods. The percentage of EGFP-positive cells increased in a dose- and time-dependent manner (Figure 2(a)). Importantly, the same stimulatory effect of these compounds was also found in the Jurkat-based postintegrative latency model 2D10 (Figure 2(b)), which is another well-characterized latency model harboring almost the complete HIV genome with only the nef gene replaced by that encoding EGFP.

To make sure whether the results measured by FACS were on account of the GFP expression, quantitative RT-PCR with primers that hybridize to a distal portion of the GFP gene and western blotting with anti-GFP were performed in J-Lat A2 and 2D10 cells, as indicated in Figures 2(c) and 2(d)). Utilizing these two experimental methods could attain the consistent points that these four compounds reverse latent HIV provirus at the protein and mRNA levels.

### 3.3. Polyphenol Extracts from Grape Seeds, Apple, Pomegranate, and Bilberry Predominantly Have the Effects Which Stimulate Tat-Independent HIV-1 Transcription

**Figure 3:**
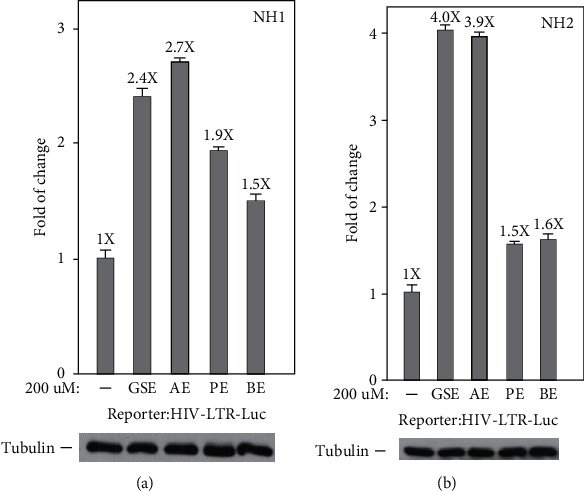
Polyphenol extracts from grape seeds, apple, pomegranate, and bilberry predominantly have the effects which stimulate Tat-independent HIV-1 transcription. The HeLa-based NH1 cells expressing no Tat (a) and NH2 cells stably expressing Tat-HA (b), with both containing an integrated HIV LTR-luciferase construct, were treated with polyphenol extracts from grape seeds, apple, pomegranate, and bilberry for 24 h. Whole cell extracts (WCE) were examined for the contained luciferase activities and cell tubulin protein as a reference by western blotting. The error bars in all panels represent mean ± SD from three independent experiments.

To confirm that these four agents' stimulation of the production of GFP mRNA and protein in latently infected Jurkat cells merely relied on the activation of the HIV 5′-LTR, but not any other unrelated viral or nonviral sequences in the integrated HIV elements, we inspected the function of these four agents that were treated in the NH1 cell line including an integrated luciferase reporter gene that is driven solely by the HIV 5′-LTR [19, 26]. The results in Figure 3(a) show that polyphenol extracts from grape seeds, apple, pomegranate, and bilberry (200 *μ*M for 24 h) increased the LTR-driven luciferase expression in NH1 cells (2.4-, 2.7-, 1.9-, and 1.5-fold). Besides, in the NH2 cell line, another HeLa-based cells expressing the HIV-1 Tat protein were stably transfected into these cells; four agents were found to activate the HIV LTR more potently in this isogenic cell line (4.0-, 3.9-, 1.5-, and 1.6-fold; Figure 3(b)). Together, these data prove that these four agents worked through the HIV-1 5′-LTR and had synergetic effects with Tat in HIV-1 transcription.

### 3.4. Polyphenol Extracts from Grape Seeds and Apple Promote the Release of P-TEFb from 7SK snRNP

**Figure 4:**
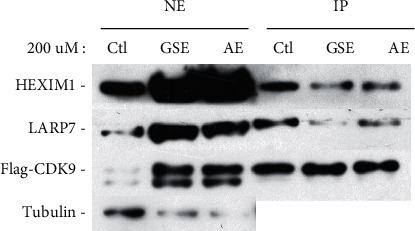
Polyphenol extracts from grape seeds and apple promote the release of P-TEFb from 7SK snRNP. HeLa-based cells that were either untreated (−) or treated (+) with polyphenol extracts from grape seeds and apple (200 *μ*M) were analyzed by western blotting for the indicated proteins. Anti-Flag immunoprecipitates (IP) derived from NE were tested by western blotting for the presence of the indicated proteins.

P-TEFb plays a key role in HIV-1 transcription, and its sequestration in the inactive 7SK snRNP has been proposed to contribute to viral latency. To confirm whether polyphenol extracts may affect the level of P-TEFb present in 7SK snRNP, we chose polyphenol extracts from grape seeds and apple and performed anti-CDK9 immunoprecipitation in HeLa cells that were treated with polyphenol extracts from grape seeds and apple. Then, we examined the association of CDK9 with two signature 7SK snRNP components HEXIM1 and LARP7 in the immunoprecipitates through western blotting. As shown in Figure 4, reduced amounts of HEXIM1 and LARP7 were found to bind to CDK9 upon the treatment with polyphenol extracts from grape seeds and apple. These results indicated that the treatment caused the disassociation of P-TEFb from the 7SK snRNP.

## 4. Discussion

Up to now, HIV/AIDS is still difficult to overcome for human health. Although HARRT has been regarded as the most effective way to treat HIV infection and extend the patients' lives, it was unable to eliminate the latent HIV reservoirs and the high cost of HARRT cannot but make us face a reality. Under the circumstances, a novel strategy, called “shock and kill” which combined effective latency-reversing agents without significantly impacting the host cell growth and function with HARRT, was proposed to cure infected patients of HIV/AIDS [8, 15–17]. Unfortunately, the currently available latency activators have all shown high toxicity and/or poor clinical outcomes [46].

Nowadays, dietary therapy has been used not only in the treatment of the disease but also in the application of some health care products which has provided great benefits for human health. If dietary therapy and chemotherapy are together used in the treatment of HIV infection, it is perhaps an effective way. To achieve this goal, we found that polyphenol extracts from grape seeds, apple, pomegranate, and bilberry have been identified and shown to facilitate latent HIV transcription and predominantly have synergetic effects with Tat protein. In addition to four extracts, the other similar polyphenol-rich agents as indicated in Table 1 have a light increase in the LTR-driven GFP expression in J-Lat A2 cells. However, ginger extraction put up a strong activity, and others have little effect on 2D10 cells.

It is unknown what kind of mechanism these polyphenol extracts work, and combining them with chemotherapeutics to be utilized in a clinical setting is still poorly investigated. The efficiency of extracts which are in combination with HARRT is subjected to evaluation in the treatment of HIV/AIDS because food can also affect the drugs' absorption and the polyphenolic content of these extracts is not very high, so that it also forms a facing problem. Although not all the polyphenolic extraction contains an activity that reverses HIV latency and they may not necessarily be the eventual drug used clinically for waking up latent proviruses and curing HIV/AIDS, their identification and characterization in the present study serve as an important proof of concept that dietary therapy may be joined in the combinatorial treatment.

## Figures and Tables

**Table 1:** Polyphenol extracts from grape seeds, apple, pomegranate, and bilberry facilitate latent HIV transcription.

Agents (200 *μ*M)	GFP-positive cells (%) (J-Lat A2 cells)	GFP-positive cells (%) (2D10 cells)
DMSO	0.6	7.5
Litchi extract	1.2	8.4
Garlic extract	1.0	9.6
Chinese yam extract	1.3	7.5
Grape seed polyphenolic extract	29.6	56.4
Mulberry extract	1.3	6.5
Cinnamon extract	1.4	8.1
Bitter gourd extract	1.6	7.7
Hawthorn extract	1.5	6.3
Apple polyphenolic extract	24.8	60.7
Broccoli extract	1.4	8.9
Pomegranate polyphenolic extract	10.2	30.1
Chrysanthemum extract	1.6	7.0
Chinese date extract	1.6	10.6
Emblic leafflower fruit extract	1.6	11.2
Wolfberry extract	1.8	8.1
Mushroom extract	1.7	8.1
Mint extract	1.7	7.5
Walnut extract	1.6	7.8
Papaya extract	2.0	8.0
Kelp extract	1.8	9.5
Ginger extract	1.6	28.2
Bilberry polyphenolic extract	12.5	15.8
Prostratin-2.5 *μ*M	58.7	93.7

## Data Availability

The research data used to support the findings of this study are included within the article.
